# Structural, morphological, electrochemical, and supercapacitance capability of NiCoMoO_4_-doped MoS_2_ nanoplates

**DOI:** 10.1039/d5ra01168j

**Published:** 2025-05-15

**Authors:** Kazem Karami, Elahe Ziaee Jazi, Nasrin Jamshidian, Alireza Allafchian

**Affiliations:** a Department of Chemistry, Isfahan University of Technology Isfahan 84156/83111 Iran; b Research Institute for Nanotechnology and Advanced Materials, Isfahan University of Technology Isfahan 84156/83111 Iran

## Abstract

In this study, spinel-nanostructured NiCoMoO_4_ (NCMO) was synthesized using a hydrothermal method and subsequently doped on molybdenum disulfide plates through the same process. The synthesized compounds were characterized using different instrumental methods. Spinel-nanostructured NCMO exhibited a regular shape and a flower-like morphology with a monoclinic crystal phase and an average 452 nm crystallite size. The NCMO@MoS_2_ nanocomposite showed the same morphology with 236 nm crystallites. The thermal gravimetry analysis showed that this compound has good thermal stability, losing only 41.70% of its weight up to 600 °C. Also, the use of the NCMO@MoS_2_ nanocomposite as an asymmetric supercapacitor was investigated. The results showed that at a current density of 50 mA g^−1^, the NCMO@MoS_2_ electrode achieved a maximum capacitance of 571.42 F g^−1^, along with a power density of 1785.5 W kg^−1^ and an energy density of 83.19 Wh kg^−1^, demonstrating its effective functioning as a supercapacitor. Also, the obtained charge transfer resistance (*R*_CT_) and series resistance (*R*_S_) from electrochemical impedance spectroscopy were 1.40 Ω and 1.02 Ω, respectively. These characteristics confirm the ideal supercapacitor behavior. In addition, the NCMO@MoS_2_ electrode showed good cyclic stability after 2000 cycles and maintained 88% of its initial capacity. An NCMO@MoS_2_//AC asymmetric supercapacitor was created for practical use and showed a capacitance of 75 mF g^−1^, along with a power density of 900 W kg^−1^ and an energy density of 0.015 Wh kg^−1^ at a current density of 9 mA g^−1^.

## Abstract 2

This article investigates the structural, morphological, and electrochemical properties of NiCoMoO_4_-doped MoS_2_ nanoplates, highlighting their enhanced supercapacitor performance and potential applications in energy storage devices.

## Graphical abstract

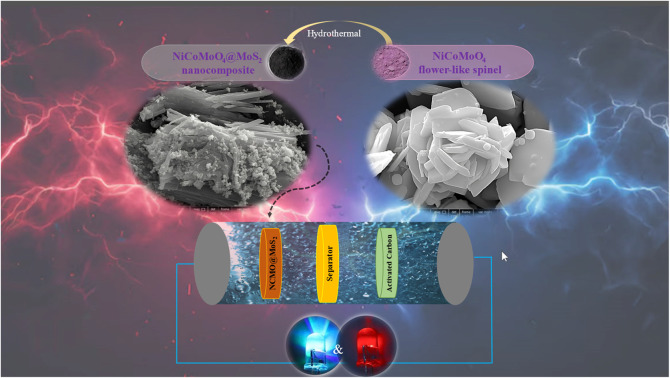

## 1. Introduction

Today, the need for clean energy sources is felt to preserve natural resources and regulate global energy consumption. Energy storage devices are used in various fields, such as electric vehicles, hybrid vehicles, and portable electronics, including electrical devices, supercapacitors, batteries, and fuel cells.^1^

Electrochemical energy storage using capacitors and batteries plays a significant role in electric vehicles and portable electronics. Although energy storage devices are similar in terms of having two electrodes, including a cathode and an anode in an electrolyte and supplying energy at the common electrode–electrolyte boundary, they have slight differences in the mechanism of energy storage and conversion.^2,3^ Electrochemical capacitors (supercapacitors) have received much attention due to their higher power, longer life cycle, excellent specific power, and fast charge–discharge capability.^4^

Supercapacitors are classified into three fundamental types including (a) electrochemical double-layer capacitors (EDLC), (b) pseudo-capacitors (PCs), and (c) hybrid supercapacitors that use both mechanisms.^5^ Hybrid supercapacitors try to reduce the relative disadvantages of EDLCs and pseudo-capacitors to achieve better performance characteristics.^6,7^

One group of structures widely used as supercapacitors are transition metal spinels. These compounds belong to a large family of minerals with the general formula AB_2_X_4_, where A is a divalent cation such as magnesium, manganese, iron, cobalt, nickel, copper, zinc, *etc.*, B is a trivalent cation such as aluminum, manganese, iron, cobalt, nickel, and X is either oxygen or sulfur.

Among the different transition metal spinels studied for supercapacitor working electrodes, nanostructured transition metal oxides with mixed metals are particularly unique and noteworthy due to their mechanical and chemical contexts stability, the variable oxidation states caused by the half-filled d substrate, which results in a variety of magnetic, electrical, magneto-optical, and optoelectronic properties.^8,9^

These nanostructures exhibit a high surface area along with a high surface-to-volume ratio and a porous structure compared to their bulk counterparts, which enhances the efficient use of these oxides in energy storage applications.^10^ For practical energy storage, these compounds must exhibit some critical characteristics, including high electrical conductivity, variable oxidation states without observed phase change over a wide potential range, and fast redox reactions facilitated by the exchange of ions between layers of matter.^11^ Several possible redox sites and oxidation states present in polymetallic systems, as well as more significant concentrations of active sites, contribute to increasing the rate of charge release.^12^

Transition metal molybdates (AMoO_4_) have attracted considerable scholarly interest due to their exceptional electrochemical properties, which are significantly influenced by the intrinsic characteristics of divalent metal cations (A^2+^). A series of molybdate compounds, including NiMoO_4_, CoMoO_4_, ZnMoO_4_, MnMoO_4_, and FeMoO_4_. Trimetallic Ni–Co–Mo oxides have become essential materials in various electrochemical applications, particularly in energy storage. The ionic radii of Ni^2+^ and Co^3+^ are comparable to that of Mo^6+^(as a high-valence non-3d transition metal ion), leading to synergistic interactions among these three metals that enhance their electrochemical performance. Consequently, Ni–Co bimetallic molybdate spinels (NiCoMoO_4_) demonstrate unique attributes and superior electrochemical properties when compared to their counterparts, NiMoO_4_ and CoMoO_4_.

MoS_2_ is one of the transition metal chalcogenides with a layered structure and a high surface area to volume ratio, and is one of the widely studied nanomaterials in the field of energy storage. The electron correlation between Mo atoms is responsible for enhancing electronic transfer and conduction in MoS_2_. The covalent interaction between Mo and S atoms and the van der Waals interaction between their layers make it useful in various electronic applications.^13^ By adjusting the number of layers or the thickness of the structure to multiple layers, these nanoplates can have better optical and electronic properties due to the change in band structure and electronic arrangement.^14^

The synergistic effects between NiCoMoO_4_ and MoS_2_ nanosheets promote charge delocalization and enhance the supercapacitor performance. In this study, a novel type of spinel nanostructure doped on molybdenum disulfide nanoplates was synthesized, and the supercapacitor activity of the final nanocomposite was investigated using different electrochemical methods.

## 2. Experimental

### 2.1. Materials

Nickel nitrate hexahydrate (Ni(NO_3_)_2_·6H_2_O), Cobalt dinitrate hexahydrate Co(NO_3_)_2_·6H_2_O, potassium hydroxide (KOH), ammonium fluoride (NH_4_F), *N*-methyl-2-pyrrolidone (C_5_H_9_NO), hydrochloric acid (HCl), polyvinylidene fluoride (–(C_2_H_2_F_2_)_*n*_–) were purchased from Merck Corporation and used without further purification. In addition, sodium molybdate dihydrate (Na_2_MoO_4_·2H_2_O), thiourea (CH_4_N_2_S), carbon black, thioacetamide (C_2_H_5_NS), and polyvinyl alcohol (–[CH_2_CH(OH)]_*n*_–) were purchased from Sigma-Aldrich Corporation and used without further purification.

### 2.2. Synthesis of spinel-nanostructured NCMO

Spinel-nanostructured NCMO was prepared with a slight change from the method presented in previous articles.^15^ Ni(NO_3_)_2_·6H_2_O (290 mg, 1 mmol), Co(NO_3_)_2_·6H_2_O (290 mg, 1 mmol), and Na_2_MoO_4_·2H_2_O (242 mg, 1 mmol) were mixed with 50 mL of deionized water to obtain a lilac color suspension.

**Fig. 1:**
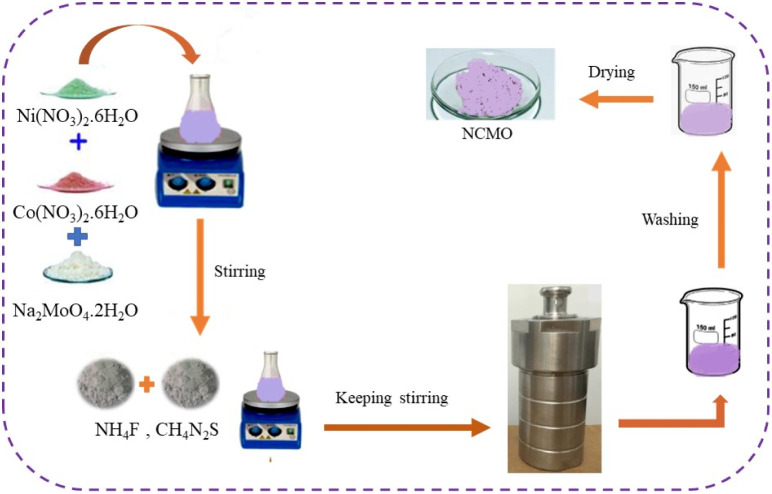
A graphical illustration of the synthesis of spinel-nanostructured NCMO.

The mixture was stirred until complete dissolution. Then CH4N2S (7.612 g, 100 mmol) and NH4F (370 mg, 10 mmol) were added to it and stirred for 1 h. The resulting mixture was poured into an autoclave and kept in an oven at 150 °C for 16 h. After cooling at room temperature, it was centrifuged for 15 minutes (6000 rpm). The final product was washed 3 times with deionized water and ethanol and ultimately dried at 70 °C for 24 h and at 200 °C for 5 h (Fig. 1).

### 2.3. Synthesis of NCMO@MoS_2_ nanocomposite

**Fig. 2:**
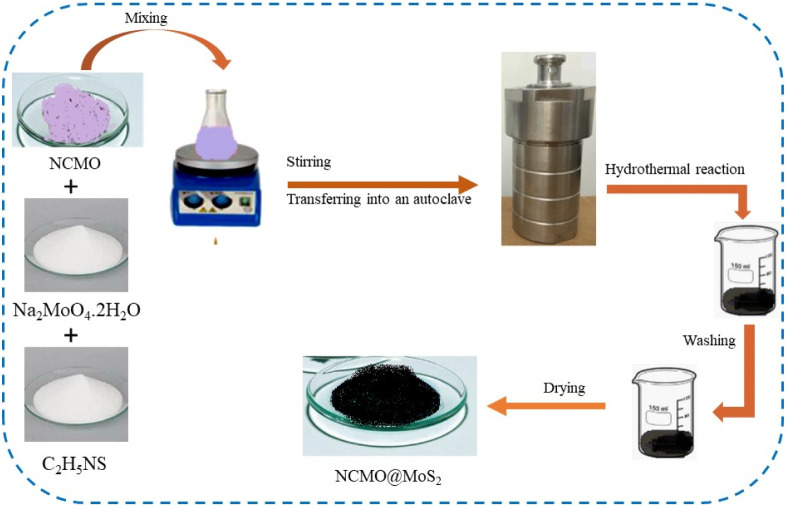
A graphical illustration of the synthesis of NCMO@MoS_2_ nanocomposite.

Based on the method presented in previous articles, with some modifications.^16^ spinel-nanostructured NCMO (2 mmol) obtained in the last step, Na2MoO_4_·2H_2_O (2 mmol), and C_2_H_5_N_S_ (20 mmol) were mixed with 60 ml of deionized water and stirred for 30 min. Then, the mixture was put in an autoclave and kept in an oven at 200 °C for 24 h. After cooling at room temperature, the black precipitate was collected and washed 3 times with deionized water and ethanol. The final precipitate was centrifuged for 15 minutes (6000 rpm) and dried at 70 °C for 48 h (Fig. 2).

### 2.4. Pre-treatment of nickel foam

First, nickel foam cut into a square shape (dimensions 1 × 1 cm) was placed in a 3 M HCl solution for 30 s and then in a mixture of ethanol and water (50/50, v/v%) in an ultrasonic bath for 3 min. Then, it was washed with distilled water, immersed in pure acetone for 30 s, and finally dried in an oven at 60 °C for 24 h.

### 2.5. Preparation of the working electrode

Nickel foam was used to fabricate the working electrode. At first, a combination of NCMO@MoS_2_ nanocomposite (85 wt%), carbon black (10 wt%) as a conductive agent, and polyvinylidene fluoride (PVDF) (5 wt%) as a binder were mixed in one drop of *N*-methyl-2-pyrrolidone. The resulting paste was dissolved with acetone (50 L) and then loaded onto the prepared nickel foam. Eventually, it was dried in an oven at 65 °C for 24 h.

### 2.6. Preparation of PVA-KOH gel

In the two-electrode system, PVA-KOH electrolyte gel was used instead of KOH liquid electrolyte to perform electrochemical tests. Due to having characteristics such as high hydrophilicity, high conductivity, compatibility with electrode and electrolyte materials, and high thermal stability, polyvinyl alcohol (PVA) can be used as a suitable candidate in the preparation of gel in supercapacitors.

To prepare the PVA-KOH electrolyte gel at room temperature, PVA powder (3 g) was slowly added to 30 ml of water for 5 consecutive hours and stirred to obtain a transparent gel with high viscosity. Then, 3 M KOH solution (5 ml) was added dropwise for two successive hours to 10 ml of the prepared gel on a magnetic stirrer to obtain PVA-KOH gel. KOH salt was used as an electrolyte. Finally, some PVA-KOH gel was placed between the working electrode, the separator, and the carbon electrode to become a two-electrode structure supercapacitor.

### 2.7. Structural characterization

FT-IR spectroscopic analyses were performed using the Plus-JASCO.600 device (JAPAN) and KBr tablets, scanning from 400 to 4000 cm^−1^. To determine the crystal structure and existing phases of the synthesized samples, X-ray diffraction analysis was performed using DW-XRD-Y3500C High Precision XRD Diffractometer at 30 kV with Cu Kα radiation (*λ* = 1.5418 Å) (China) with step time = 1 s, step size = 0.05° and 2*θ* = 10–90°. Differential scanning calorimeter analysis device model TA-1 made by Pishtaz Equipment Company (IRI). The morphological properties of the powders were investigated using the Field emission scanning electron microscope (FESEM) model QUANTA FEG-450 made by FEI (USA). The compositions were analyzed using an energy dissipative detector (EDS) Octane Elite model made by AMETEK (UK), coupled with the SEM.

### 2.8. Electrochemical analyses

Evaluation of electrochemical performance through cyclic voltammetry, galvanostatic charge–discharge, and electrochemical impedance spectroscopies was performed using an AUTOLAB PGSTAT device (Germany).

## 3. Results and discussion

### 3.1. Spectral characterization

#### 3.1.1. FT-IR spectroscopy

**Fig. 3:**
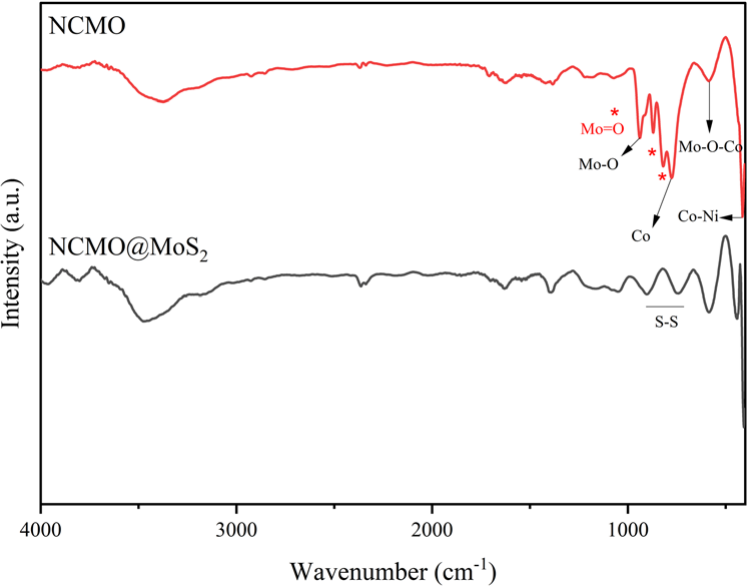
FT-IR Spectra of spinel-nanostructured NCMO and (black) NCMO@MoS_2_ nanocomposite.

FT-IR spectra of spinel-nanostructured NCMO and NCMO@MoS_2_ nanocomposite were shown in Fig. 3. In the red spectrum, the absorption bands at 937 and 590 cm^−1^ correspond to Mo–O and Mo–O–Co stretching vibrations, respectively. The narrow band at 414 cm^−1^ is attributed to the Co–Ni vibration.^17^ Also, the absorption bands at 1068, 836, and 879 cm^−1^, which are characteristic of tetrahedral MoO_4_ groups, confirm the formation of molybdenum tetrahedral coordination compounds. The absorption band observed at about 779 cm^−1^ is related to the bending modes of vibration of cobalt.^18^ In the black spectrum, the absorption band at 455 cm^−1^ is related to the Mo–S bond, and the bands observed in the region of 1200–700 cm^−1^ are associated with S–S vibrations. Also, characteristic peaks of 443, 574, 744, 904, and 1043 cm^−1^ are visible which were transferred to higher frequencies due to surface interaction.^17^ The broad absorption band in the range of 3400–3600 cm^−1^ is visible in all three spectra and is related to the O–H stretching vibration of coordinated/entrapped water in synthesized compounds.

#### 3.1.2. X-ray powder diffraction (XRD)

**Fig. 4:**
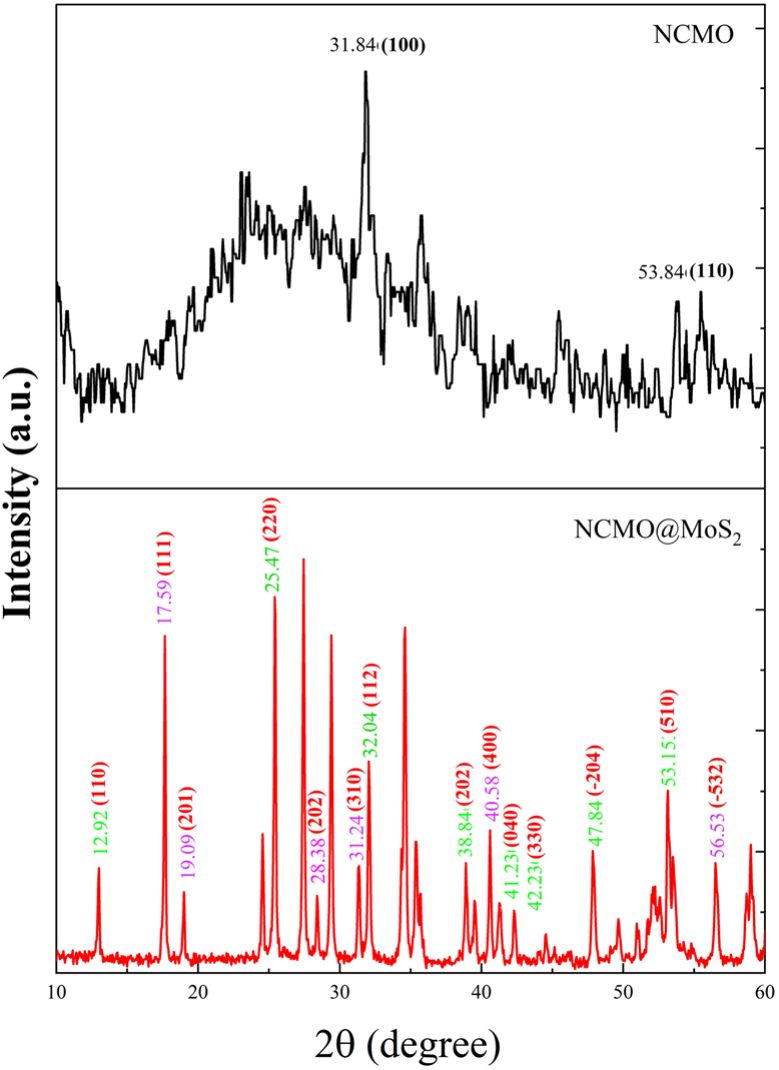
XRD patterns of spinel-nanostructured NCMO and NCMO@MoS_2_ nanocomposite.

XRD analysis was used to confirm the crystalline structures of the synthesized particles and determine diffraction parameters. Their X-ray diffraction patterns are shown in Fig. 4. For spinel-nanostructured NCMO, the observed pattern indicates a monoclinic phase.

In this diffraction pattern, two distinct crystalline phases are observed, corresponding to the normal and inverted spinel structures of NiCoMoO_4_. This confirms the successful incorporation of both spinel configurations, with both phases coexisting in the material, and the successful combination of NiMoO_4_ and CoMoO_4_ structural plates. For NiMoO_4_, the peaks at 2*θ* = 12.92°, 25.47°, 32.04°, 38.84°, 41.23°, 42.23°, 47.84°, and 53.15° correspond to (110), (220), (112), (202), (040), (330), (204), and (510) lattice planes, respectively.^19^ Additionally, in the case of CoMoO_4_, the peaks at 17.95°, 19.09°, 28.38°, 31.24°, 40.58°, and 56.53° correspond to (111), (201), (202), (310), (400), and (−532) lattice planes, respectively.^20–22^

For NCMO@MoS_2_ nanocomposite, the peaks at 31.84° and 53.84° are related to (100) and (110) lattice planes, respectively. This pattern indicates the amorphous structure of this nanocomposite, which is well-proven by the FE-SEM images. The broadening of the peaks compared to NCMO crystals indicates that the final product consists of monolayer or multilayer MoS_2_ plates.^23^

The crystallite size (*L*), the density of dislocation (*d*), the micro-strain (*e*), and the values of atomic plane spacing (*d*) of all samples were calculated using eqn (1)–(4), respectively:^24^1
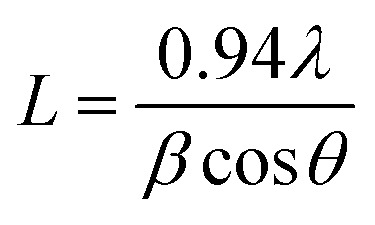

2 *δ* = 1/*L*^2^

3
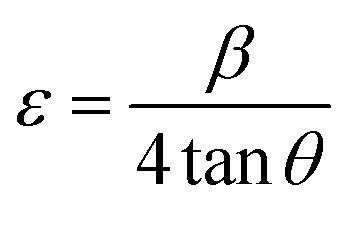
4
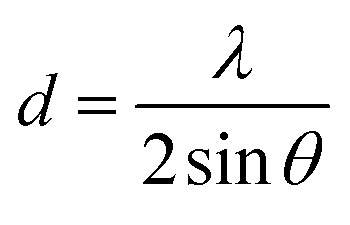
where *l* = X-ray wavelength (1.5406 Å), *b* = diffraction peak's FWHM (full width at half-maximum), and *q* = diffraction angle. The calculation results are shown in Table 1. Compared to Spinel-nanostructured NCMO, the NCMO@MoS_2_ nanocomposite exhibits a greater dislocation density, smaller crystallite size, and atomic plane spacing, all validating distortion. Consequently, lattice defects occur, resulting in more active sites with an enhanced surface area and increased charge transfer rate for active materials.^25–27^

**Table 1:** Calculated diffraction parameters for spinel-nanostructured NCMO and NCMO@MoS_2_ nanocomposite

Synthesized particle	*β* (radian)	*L* (nm)	*d* (Å)	ε	*δ* × 10^−6^ (nm^−2^)
NCMO	0.31	447	5.02	0.50	5.00
	0.44	452	3.501	0.35	4.89
	0.56	459	2.79	0.27	4.74
NCMO@MoS_2_	0.55	229	2.81	0.55	19.06
	0.62	232	2.51	0.49	18.57
	0.94	247	1.70	0.31	16.39

#### 3.1.3. Thermal gravimetric analysis (TGA-DSC)

**Fig. 5:**
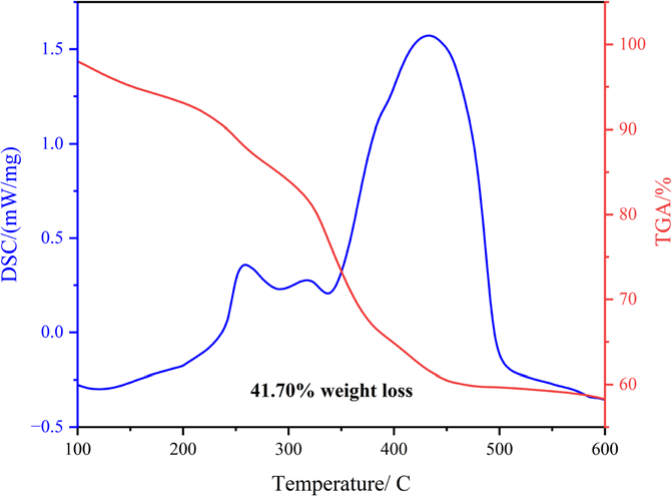
TGA (red) and DSC (blue) curves of spinel-nanostructured NCMO and NCMO@MoS_2_ nanocomposite.

In thermal gravimetry analysis (TGA), it is possible to increase or decrease the weight of the compound under the influence of temperature. Weight gain is caused by internal and surface absorption, while weight loss is caused by desorption, evaporation, destruction, and drying. As shown in the TGA curve of NCMO@MoS_2_ (Fig. 5), the process of thermal decomposition in the nitrogen-oxygen atmosphere includes three stages in the temperature ranges 100–200 °C, 250–300 °C, and 320–400 °C. The first weight loss is mainly attributed to the loss of surface adsorbed water or residual water molecules. The second weight loss is a result of the decomposition of water of crystallization, and the third stage is due to the removal of compounds such as thiourea, the destruction of sulfur compounds, the removal of organic molecules in the synthesized nanostructure, and the change of structure.

The sample weight is reduced by 41.70% during these three stages, and its weight remains almost constant above 450 °C, indicating that the final decomposed products correspond to NCMO@MoS_2_. The thermal decomposition DSC curve of NCMO@MoS_2_ in the O_2_/N_2_ atmosphere shows three peaks and one shoulder. The first stage at 220–280 °C is related to the dehydration of hydroxide and conversion to oxide. The second stage at 290–350 °C is the removal of network water molecules and the remaining hydroxide groups. The shoulder near 400 °C is due to the decomposition of NiO into metallic Ni. The last stage is a result of the destruction of the network, which is highly endothermic. This process involves complete pyrolysis and leads to phase change. According to this curve, all phases are endothermic.^28^

#### 3.1.4. Morphology and size studies

**Fig. 6:**
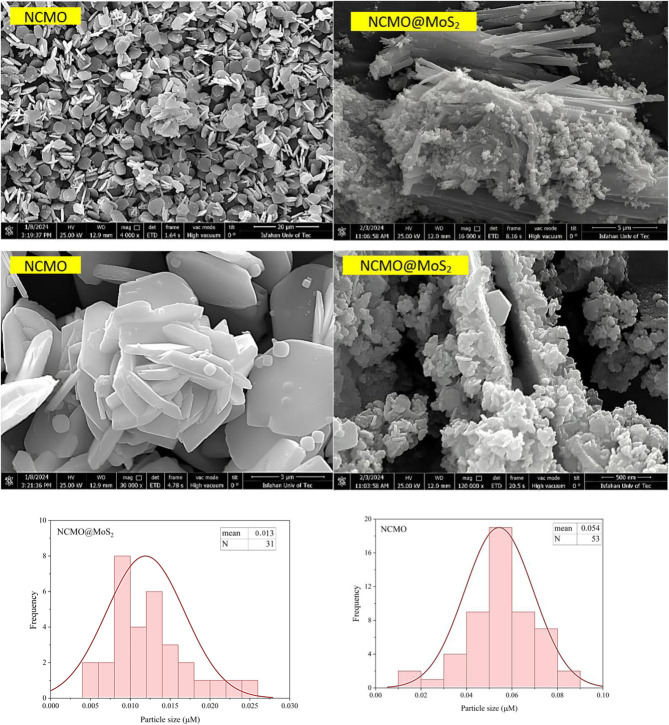
SEM images (different magnifications) and particle size distribution histogram of three-dimensional flower-like Spinel-nanostructured NCMO and NCMO@MoS_2_ nanocomposite.

The morphology and crystallinity of the synthesized compounds were studied by a field emission scanning electron microscope. According to Fig. 6, NCMO nanoparticles have a regular shape and a flower-like morphology. The average diameter of the particles in line with the corresponding histogram is 54 nm. For NCMO@MoS_2_ nanocomposite, the disorder is observed without a noticeable change in morphology. Using the corresponding histogram, the diameter of NCMO@MoS_2_ particles is 13 nm, consistent with the X-ray diffraction images.

#### 3.1.5. Elemental composition studies

**Fig. 7:**
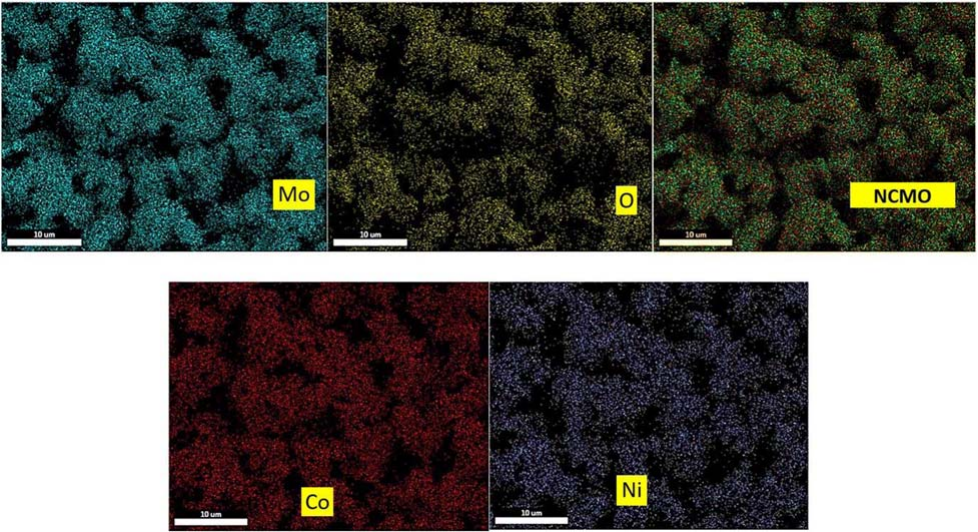
X-ray mapping analysis of Spinel-nanostructured NCMO.

**Fig. 8:**
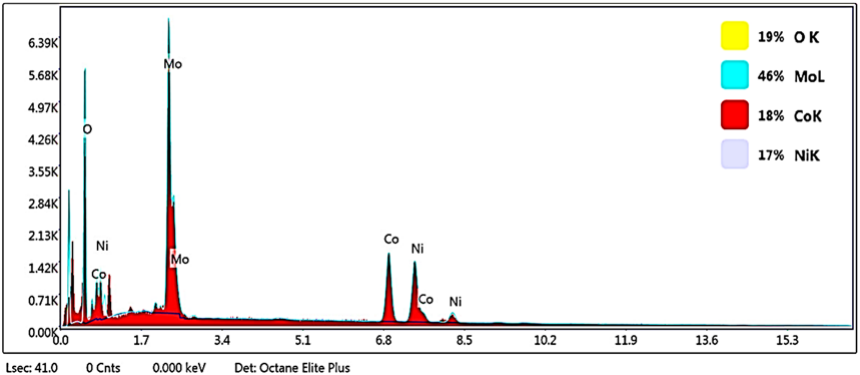
EDX analysis graph of Spinel-nanostructured NCMO, where the *X*-axis shows the energy in keV and the *Y*-axis signifies intensity count.

**Fig. 9:**
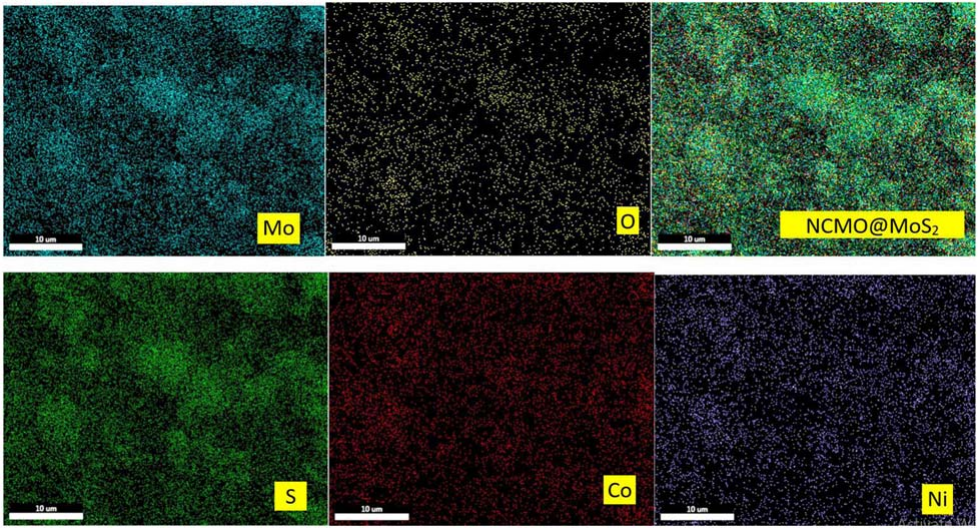
X-ray mapping analysis of NCMO@MoS_2_.

**Fig. 10:**
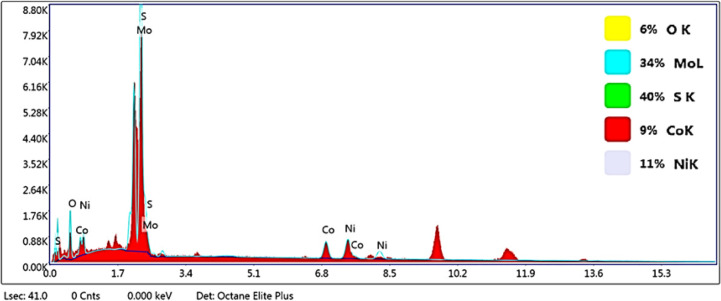
EDX analysis graph of NCMO@MoS_2_ nanocomposite, where the *X*-axis shows the energy in keV and the *Y*-axis signifies intensity count.

X-ray energy diffraction spectrometry was used to study the type of elements present in the prepared nanoparticles. In addition, the surface mapping test was utilized to check the uniform distribution of elements in them (Fig. 7, and 9). As it is apparent in the EDAX graphs (Fig. 8 and 10), the peaks related to the presence of Ni, Co, Mo, O, and S indicate the purity and successful synthesis of the prepared compounds. In addition, the weight percentages of Ni, Co, Mo, and O in NCMO were 17%, 18%, 46%, and 19%, respectively. For NCMO@MoS_2_, the weight percentages of Ni, Co, Mo, O, and S were 11%, 9%, 34%, 6%, and 40%, respectively. As evident, the elements are dispersed regularly and uniformly, showing the lack of agglomeration.

### 3.2. Electrochemical studies

#### 3.2.1. Cyclic voltammetry

**Fig. 11:**
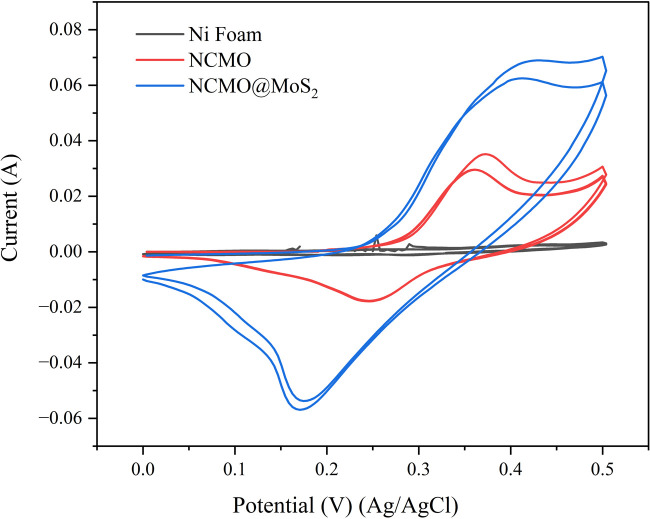
Cyclic voltammetry curves at a constant scanning speed of 120 mV s^−1^ and the applied potential window 0–0.5 V.

The electrochemical performance of the electrode prepared with NCMO@MoS_2_ and NCMO was evaluated using a three-electrode configuration system in 3 M KOH solution at room temperature. So, Pt and Ag/AgCl were used as fixed electrodes that played the role of counter (auxiliary) and reference electrodes, respectively. Spinel-nanostructured NCMO and NCMO@MoS_2_ nanocomposite were used as working electrodes. Cyclic voltammograms, with a constant scanning speed of 120 mV s^−1^ and the applied potential window between 0 and 0.5 V shown in Fig. 11.

As can be seen, there is a noticeable difference between the voltammograms of nickel foam electrodes and synthesized compounds. Nickel foam alone has a small current and indicates the insignificant contribution of the support in charge storage. The NCMO nanoparticles have a smaller surface area and current.^29^ In contrast, the NCMO@MoS_2_ nanocomposite shows considerable improvement in surface area and capacitance. It can be concluded that the MoS_2_ nanoplates can improve the electrochemical properties of NCMO nanoparticles. It is noteworthy that for spinel-nanostructured NCMO and NCMO@MoS_2_ nanocomposite, increasing the scan speed leads to redox peaks moving to higher and lower potentials, respectively. Also, the potential difference between oxidation peaks and reduction peaks increased with increasing scanning speed.^30^

**Fig. 12:**
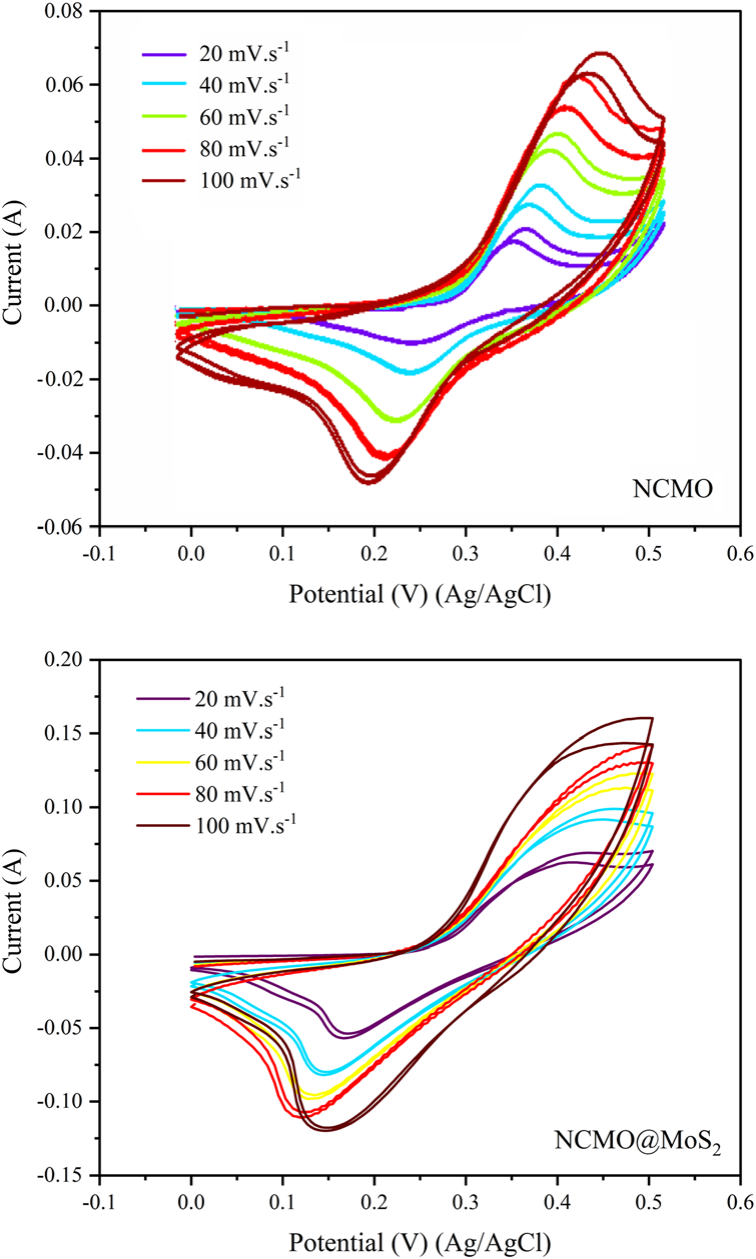
Cyclic voltammogram of spinel-nanostructured NCMO and NCMO@MoS_2_ nanocomposite at various scan rates and the applied potential window 0 to 0.5 V.

Fig. 12 shows the cyclic voltammogram for synthesized compounds at the scanning speed of 20 to 100 mV s^−1^. The capacitance of the electrodes is calculated using eqn (5):5
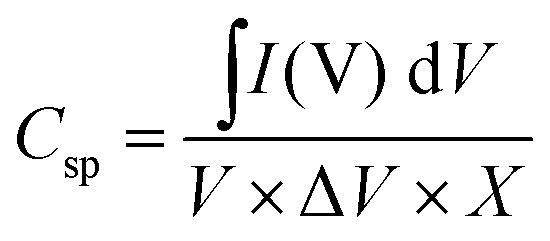
where *C*_sp_ (F g^−1^) is the specific capacitance, *V* (V s^−1^) is the scanning speed, *X* (g) is the amount of active material loaded on the electrode surface, *I* (A) is the applied current density, and Δ*V* (v) is the potential window. The calculated capacities for the NCMO nanoparticles at the scanning speed of 20, 40, 60, 80, and 100 mV s^−1^ are 57.45, 49.88, 45.13, 33.74, and 31.34 F g^−1^, respectively. The values for NCMO@MoS_2_ at scanning speeds of 20, 40, 60, 80, and 100 mV s^−1^ were 182.85, 140.41, 116.97, 101.55, and 87.54 F g^−1^, respectively. The oxidation-reduction peaks of CV curves are due to the faradaic reactions of Ni and Co ions associated with OH^−^ anions. These reactions are listed as follows (M represents Ni, Co, and Mo ions):3[M(OH)_3_]^−^ ⇔ 3MOOH + 3H_2_O + 3e^−^CoOOH + OH^−^ ⇔ CoO_2_ + H_2_O + e^−^CoOOH + H_2_O + e^−^ ⇔ Co(OH)_2_ + OH^−^

#### 3.2.2. Chronopotentiometry

**Fig. 13:**
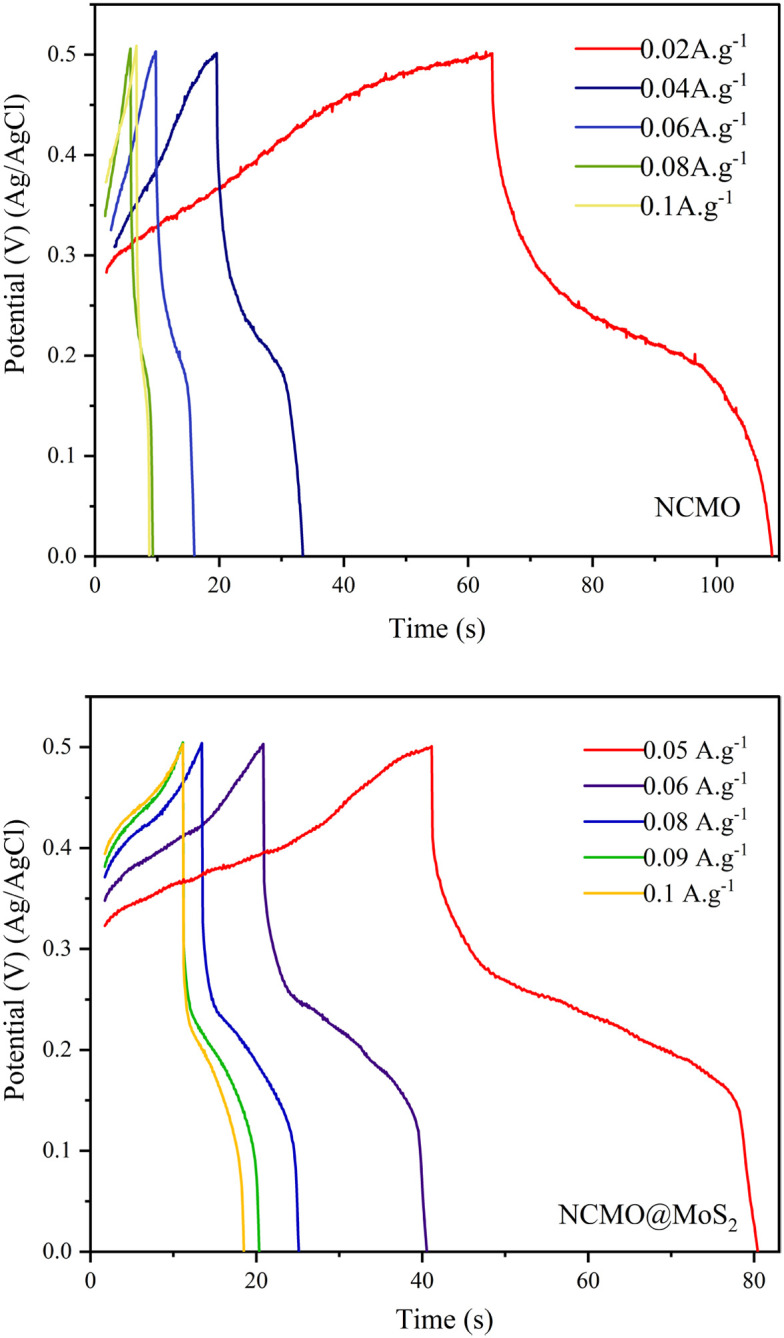
Galvanostatic charging–discharging curves of spinel-nanostructured NCMO and NCMO@MoS_2_ nanocomposite at different current densities.

This technique involves applying a specific positive current for charging, followed by a negative current for discharging at specific time intervals Fig. 13.

**Fig. 14:**
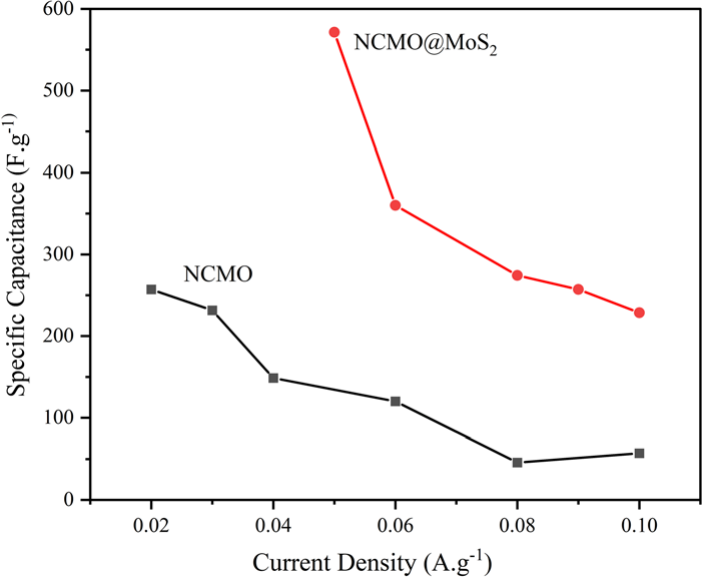
Specific capacitance at different current densities for Spinel-nanostructured NCMO and NCMO@MoS_2_ nanocomposite.

As can be seen in Fig. 14, the increase in the galvanostatic charge/discharge time of the NCMO@MoS_2_ nanocomposite had a more significant change in discharge time at the current densities of 60, 80, and 100 mA g^−1^, indicating a higher capacity of this electrode compared to NCMO. These curves correspond to the voltammograms of the NCMO@MoS_2_ nanocomposite, which confirms its supercapacitor behavior. The created graphs show that the charge–discharge processes follow faradaic and pseudo-capacitive mechanisms.

It is also clear that with increasing current density, the discharge time decreases. This is because the charge–discharge process takes place through the absorption–desorption of charge (cation) at the locations of the electrode surface or through the penetration of the charge into the electrode tissue.

At low scanning speeds, the charge has enough opportunity to penetrate the electrode surface and the electrode tissue. Both the surface and the electrode tissue participate in the charge–discharge process. While at high scanning speeds, the charge only covers the electrode surface and doesn't have enough opportunity to penetrate the electrode tissue. So, less capacity is seen at high scanning speeds.^31^

Also, it can be seen that in both samples, there is an ohmic drop, which is the resistance of the ionic electrolyte between the reference electrode's tip and the working electrode's surface.

To calculate the specific capacity from the chronopotentiometry curves, eqn (6) was used, and the results were presented in Fig. 14.6
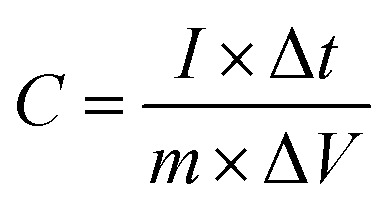
where *C* (F g^−1^) is the specific capacitance, *I* (A) is the current, Δ*t* (s) is the discharge time, *m* (g) is the amount of active material loaded on the electrode surface, and Δ*V* (v) is the potential window.^32^

As observed, the capacity of spinel-nanostructured NCMO and NCMO@MoS_2_ nanocomposite decreases with increasing current density, but the overall capacity of NCMO@MoS_2_ is nearly twice that of NCMO. The decrease in capacity is more gradual in NCMO, while in the NCMO@MoS_2_ sample, there is a sharp initial decrease followed by a gentler decline.

According to the charge–discharge curve for NCMO in the potential window of 0 to 0.5 V in the current densities of 0.02, 0.03, 0.04, 0.06, 0.08, and 0.1 A g^−1^ shows that the sample capacity decreases to 257, 231, 148, 120, 45, and 57 F g^−1^, respectively. For NCMO@MoS_2_ in the current densities of 0.05, 0.06, 0.8, 0.09, and 0.1 A g^−1^, capacities are 571, 360, 274, 257, and 228 F g^−1^, respectively.

The decrease in charge and discharge time with increasing current density is due to the decrease in effective interactions in the electrochemical active sites.

**Fig. 15:**
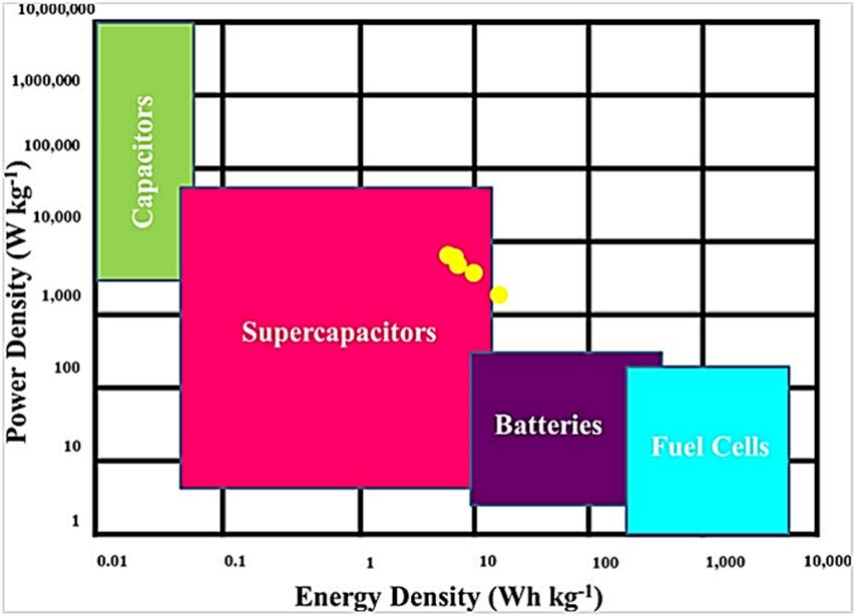
Ragone plot for NCMO@MoS_2_ electrode (power density *vs.* energy density).

Energy density and power density are crucial factors in determining the performance of the prepared electrode. The ragone plot illustrates the comparison of these two critical factors at different current densities. Eqn (7) and (8) are used to calculate power density and energy density:7
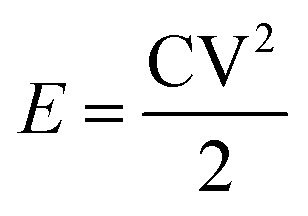
8
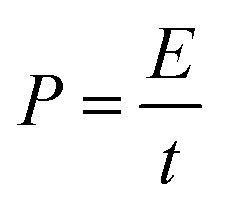
where *E*, *n* (Wh kg^−1^) is a specific energy, *C* (F g^−1^) is the specific capacitance, *V* (v) is the potential window, *P* (W kg^−1^) is power density, and *t* (s) is the discharge time. As shown in Ragone's plot for NCMO@MoS_2_ positive electrode in Fig. 15, by increasing current density from 50 to 100 mA g^−1^, the energy density decreased, but the power density increased. So, the NCMO@MoS_2_ electrode can show a power density of 1785.5 W kg^−1^ at a current density of 50 mA g^−1^.

This means that despite the high value of power density, the electroactive material can maintain its energy density value of 83.19 Wh kg^−1^, which indicates the proper performance of this material as a supercapacitor.

#### 3.2.3. Electrochemical impedance spectroscopy (EIS)

Electrochemical impedance spectroscopy (EIS) is used to evaluate electrode charge transfer performance. In the Nyquist impedance plot, the radius of the semicircle indicates the transfer of charge at the interface between the electrode and the electrolyte. The smaller radius indicates lower internal resistance, and the larger radius indicates higher internal resistance. Also, the closer the slope of the Warburg line is to 90°, the lower the charge transfer resistance (*R*_CT_), and these characteristics indicate the ideal capacitor behavior. Also, the internal resistance of the electrode (*R*_s_) can be checked in the Nyquist plot with the appearance of a semicircle in the high-frequency region.^33,34^

**Fig. 16:**
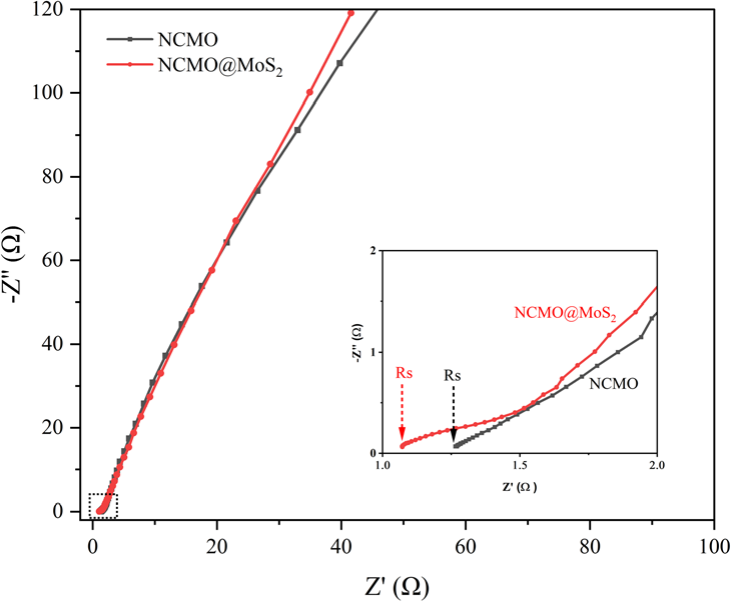
Nyquist plots of Spinel-nanostructured NCMO and NCMO@MoS_2_ nanocomposite electrodes in the frequency range from 0.1 Hz to 100 kHz at a potential of 10 mV.

Fig. 16 shows the Nyquist plots of NCMO and NCMO@MoS_2_ electrodes in the frequency range from 0.1 Hz to 100 kHz at a potential of 10 mV. As can be seen, the slope of the Warberg line for NCMO@MoS_2_ nanocomposite is closer to 90°. As a result, the KOH electrolyte creates a slight resistance, and the entry and exit of ions will be more accessible. Additionally, the obtained *R*_CT_ and *R*_s_ based on the Nyquist plot for Spinel-nanostructured NCMO are equal to 1.60 Ω and 1.22 Ω, respectively. For NCMO@MoS_2_ nanocomposite, the obtained *R*_CT_ and *R*_s_ values are equal to 1.40 Ω and 1.02 Ω, respectively. The obtained values indicate that the Spinel-nanostructured NCMO has higher internal resistance and lower conductivity than the NCMO@MoS_2_ nanocomposite. It can be due to the minimal charge storage distribution and the ohmic contact of Spinel-nanostructured NCMO with nickel foam. This result is in agreement with the parameter atomic plane spacing obtained from the XRD pattern and confirms the improvement in the performance of Spinel-nanostructured NCMO by coating it on MoS_2_ nanoplates.

### 3.3. Two-electrode system

**Fig. 17:**
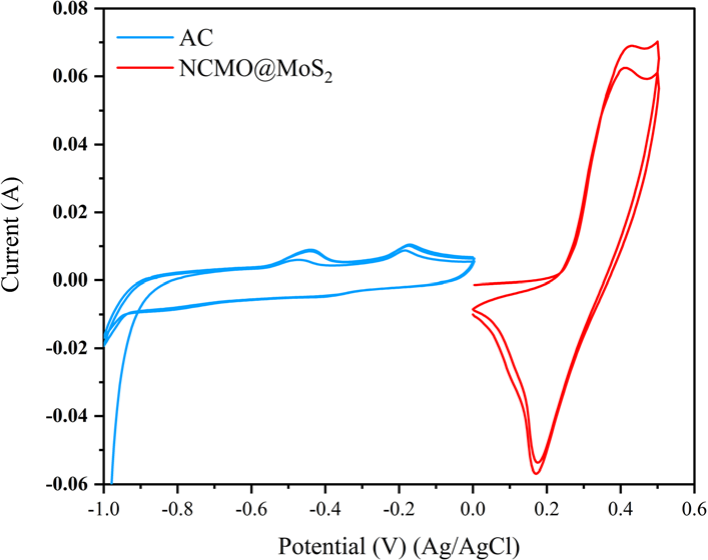
Cyclic voltammogram of NCMO@MoS_2_//AC asymmetric supercapacitor as the positive electrode and carbon black as the negative electrode at a scanning speed of 20 mV s^−1^.

For practical use, an asymmetric electrode was created. In this regard, some PVA-KOH gel was placed between the two electrodes, and filter paper was used as a separator, and compressed together. Fig. 17 displays the voltammograms of the optimized sample as the positive electrode and carbon black as the negative electrode at a scanning speed of 20 mV s^−1^. Due to the difference in materials of the positive and negative electrodes, this system is classified as asymmetric. The potential window of the carbon black electrode ranged from −1 to 0 V. The voltammogram for the negative electrode is rectangular. The shape is indeed typical for capacitive behavior, where the charge storage occurs *via* electrostatic interactions rather than through faradaic processes.

When optimizing and observing the active mass ratio in the positive and negative electrodes, a maximum potential window of 1.6 volts can be achieved. This value exceeds the potential window in the three-electrode system and in aqueous electrolytes. To avoid the release of oxygen at the working electrode, a potential window of 0.4 to 1.6 volts was selected as the optimal working potential.

**Fig. 18:**
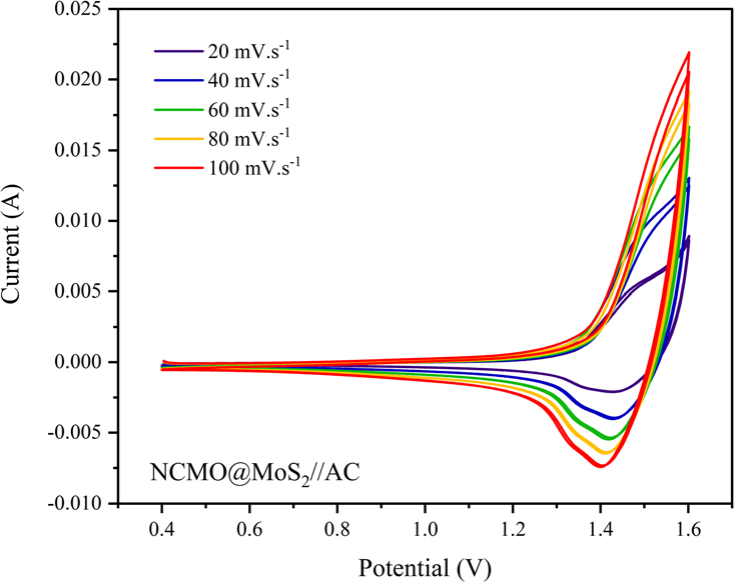
Cyclic voltammogram of NCMO@MoS_2_//AC asymmetric supercapacitor at various scan rates and the applied potential window −0.4 to 1.6 V.

As shown in Fig. 18, increasing the scanning speed leads to a larger surface area under the voltammogram, demonstrating an increase in the double-layer contribution compared to the faradaic process. Consequently, selecting a potential window of 0.4 to 1.6 V to perform electrochemical tests is an ideal and suitable decision.

**Fig. 19:**
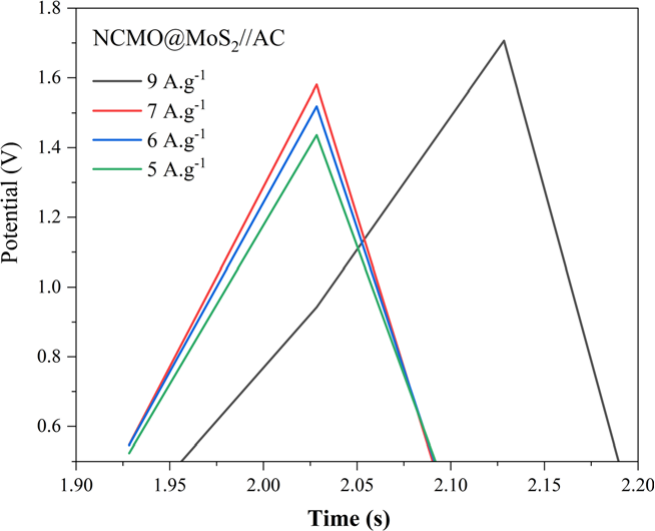
Galvanostatic charging–discharging curves of the NCMO@MoS_2_//AC asymmetric supercapacitor at different current densities.

As is evident in the chronopotentiometry curves of the NCMO@MoS_2_//AC asymmetric supercapacitor at different current densities (Fig. 19), this sample exhibits a favorable supercapacitor behavior at high currents. With the increase of current and the limitation of active sites for oxidation-reduction reactions, the electrode capacity has been reduced.

**Fig. 20:**
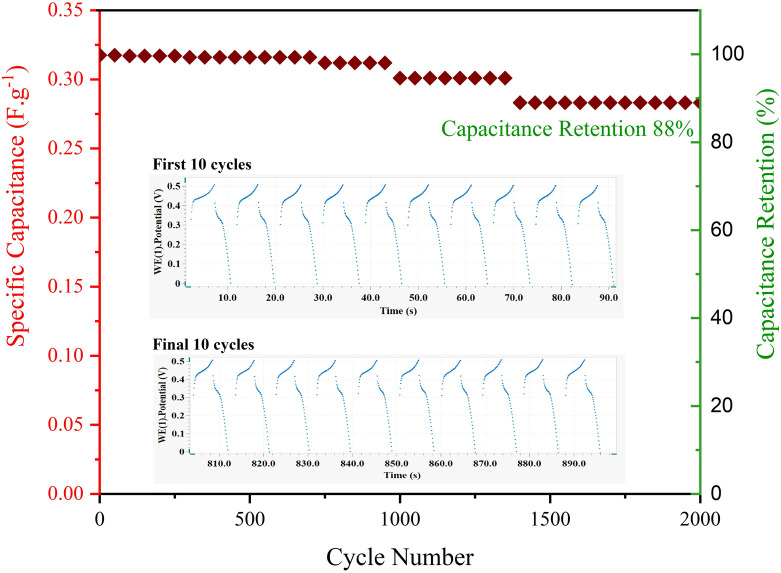
Cycling stability measurement of NCMO@MoS_2_//AC asymmetric supercapacitor at 0.102 A g^−1^ for 2000 cycles.

One of the crucial parameters in supercapacitors is cyclic stability, which shows how much of the initial capacity the supercapacitor retains after long-term use. The higher the capacity retention ability, the more industrial and practical value the manufactured supercapacitor will possess.^35^ To examine the stability of the NCMO@MoS_2_//AC asymmetric supercapacitor, it was subjected to 2000 consecutive cycles at a current density of 0.102 A g^−1^ and a voltage of 1.6 V. As can be seen in Fig. 20, after the last charge–discharge cycle, 88% of the initial capacity was recovered, indicating high cyclic life and stability of prepared supercapacitor.

**Fig. 21:**
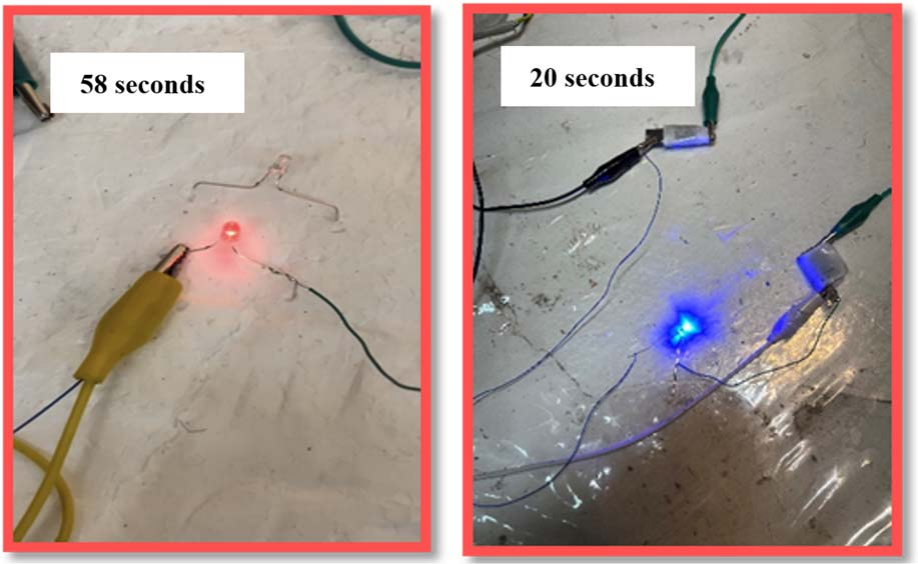
Lighting up of two commercial LEDs of various voltages (blue and red) in series with an aqueous asymmetric supercapacitor.

To demonstrate the prepared supercapacitor's practical application, two electrodes made from optimal particles were connected in series (Fig. 21). This setup turned on the blue LED lamp (3 mm, 3 V) for 20 seconds and the red LED lamp (3 mm, 2.7 V) for 58 seconds.

### 3.4. Comparison with previous studies

The synthesized NCMO@MoS_2_ nanocomposite demonstrates commendable supercapacitor performance compared to recent literature, as illustrated in Table 2. This study reveals enhanced energy and power density alongside superior specific capacitance. The increased capacity of this electrode, relative to similar studies, can be attributed to the synergistic effects of the flower-like structure of NCMO and MoS_2_ nanoplates. This structural configuration facilitates the formation of numerous electrochemically active sites that act as ion-buffer reservoirs and promote the infiltration of electrolyte ions into the matrix. Additionally, the proliferation of active sites allows for greater involvement of reactive substances in electrochemical processes. Consequently, this composite offers significant advantages as a promising candidate for high-performance electrode materials in supercapacitor applications.

**Table 2:** Comparison of electrochemical properties of spinel-nanostructured NCMO with different electrode materials for supercapacitor performance

Electrode material	NCMO/nickel foam	NCMO/CC	NCMO@MoS_2_/nickel foam
Morphology	Nanorod	Nanoplate	Nanocomposite
Specific capacitance (F g^−1^)	441	726.6	571.42
Electrolyte	KOH 6M	KOH 3M	KOH 3M
Power density (W kg^−1^)	775	800	1785.5
Energy density (Wh kg^−1^)	25.6	35.2	83.19
Cycle stability at (current density = 1 A g^−1^)	80% 1000 cycles	97.2% 3000 cycles	88% 2000 cycles
Ref.	36	37	This work

## 4. Conclusions

In this research, there was an attempt to prepare an electroactive material with optimal morphology and nanoscale for use in supercapacitors using a simple and inexpensive method. The results indicate that the unique structure of Spinel-nanostructured NCMO doped on MoS_2_ nanoplates led to achieving higher capacity and improving supercapacitor properties. Based on the results, the electrode prepared from the final nanocomposite has a capacity of 571.42 F g^−1^ at a current density of 1 A g^−1^. According to the voltammograms, at a scanning speed of 100 mV s^−1^, it showed a capacity equal to 182.88 F g^−1^. It also exhibited good cyclic stability after 2000 cycles, maintaining 88% of its initial capacity. The capacity calculated from the voltammograms was 182.88 F g^−1^ at a scanning speed of 100 mV s^−1^, and according to the chronopotentiometry calculations, its capacity equals 228 F g^−1^ at a current density of 100 mA. According to the chronopotentiometry curves, the energy density and power density at a current density of 50 mA and a capacity of 571.42 F g^−1^ were 83.19 Wh kg^−1^ and 1785.5 W kg^−1^, respectively.

Furthermore, in an asymmetric configuration, we demonstrated its practical application by illuminating the red LED lamp for 58 seconds and the blue LED lamp for 20 seconds. Overall, the experimental results in this work demonstrate that MoS_2_ enhances the performance of the synthetic particle and can be utilized as a suitable candidate for supercapacitor applications.

## Data availability

The data supporting this article have been included as part of the Supplementary Information.

## Author contributions

Kazem Karami–conceptualization, funding acquisition, methodology, supervision, writing–review & editing; Elahe Ziaee Jazi–data curation, formal analysis, investigation, software; Nasrin Jamshidian–conceptualization, formal analysis, methodology, supervision, writing–original draft, writing–review & editing; Alireza Allafchian–formal analysis, software.

## Conflicts of interest

There are no conflicts to declare.

## Supplementary Material

RA-015-D5RA01168J-s001
