# Supplementary material for: Structural, morphological, electrochemical, and supercapacitance capability of NiCoMoO4-doped MoS2 nanoplates
Source: RSC Adv. 2025 May 15;15(20):16229–39. doi: 10.1039/d5ra01168j (PMC12079647; doi:10.1039/d5ra01168j)
Supplement: RA-015-D5RA01168J-s001 [file RA-015-D5RA01168J-s001.pdf]

Supplementary Information (SI) for RSC Advances.
This journal is © The Royal Society of Chemistry 2025

Capacity calculations from CV:

$$C_{sp} = \frac{\int I(V)dV}{V.\Delta V.X}$$

| NCMO | | | | | NCMO@MoS2 | | | | |
|---|---|---|---|---|---|---|---|---|---|
| $\int I(V)dV$ | X | ΔV | V | $C_{sp}$ | $\int I(V)dV$ | X | ΔV | V | $C_{sp}$ |
| 0.004022 | 0.007 | 0.5 | 0.02 | 57.45885714 | 0.0128 | 0.007 | 0.5 | 0.02 | 182.8571 |
| 0.006982 | 0.007 | 0.5 | 0.04 | 49.86857143 | 0.019658 | 0.007 | 0.5 | 0.04 | 140.4143 |
| 0.009478 | 0.007 | 0.5 | 0.06 | 45.13285714 | 0.024565 | 0.007 | 0.5 | 0.06 | 116.9757 |
| 0.009448 | 0.007 | 0.5 | 0.08 | 33.7425 | 0.028436 | 0.007 | 0.5 | 0.08 | 101.5561 |
| 0.010972 | 0.007 | 0.5 | 0.1 | 31.34942857 | 0.030639 | 0.007 | 0.5 | 0.1 | 87.54054 |
